# Self-regulated dual-mode solar energy harvesting

**DOI:** 10.1073/pnas.2534717123

**Published:** 2026-03-24

**Authors:** Raphael Kay, Rafiq Omair, Joanna Aizenberg

**Affiliations:** ^a^Harvard John A. Paulson School of Engineering and Applied Sciences, Harvard University, Cambridge, MA 02138; ^b^Department of Chemistry and Chemical Biology, Harvard University, Cambridge, MA 02138

**Keywords:** solar harvesting, energy efficiency, photovoltaics

## Abstract

Traditional solar energy harvesters are single-mode—typically designed to convert available sunlight either into heat or electricity. However, neither energy form is continuously useful, and an optimal variant should instead be capable of autonomously toggling relative thermal and electrical yield based on need. Here, we introduce passive, dual-mode indoor solar energy harvesting by using a fluid layer trapped above a Fresnel lens as a phase-changing switch within a solar waveguide. When warm out and electricity is desired, the fluid is in its vapor phase, causing a refractive index difference with the microstructured lens beneath it to concentrate sunlight toward a solar cell. When cold out and heat is instead desired, the fluid condenses atop the Fresnel structure to reduce refractive index differences, causing sunlight to refract past the solar cell and convert to heat when absorbed indoors. Using water as a phase-changing switch, we built an exemplary system that self-regulates indoor temperature and solar cell light exposure over ambient heating and cooling cycles. Our approach is general across everyday materials and manufacturing methods and may be deployed on the surfaces of vehicles, greenhouses, and residential or commercial buildings.

## Significance

Harvesting solar energy is of fundamental importance to the green economy. However, traditional technologies are single-mode, converting sunlight to either heat or electricity. We introduce dual-mode solar harvesters that leverage phase-changing fluidic optical switches to passively toggle between converting sunlight into either heat or electricity, depending on environmental temperature. By delivering heat when cold out and electricity when warm out, dual-mode harvesters can reduce temporal demand mismatch and improve useful solar conversion efficiency.

Heat and electricity are two fundamental energy products of solar harvesting technologies. However, their usefulness varies temporally. For example, within residential, commercial, or agricultural buildings—where at least one quarter of our world’s energy is consumed (1, 2)—thermal energy is more valuable when the outside temperature is cool, and the indoor space is in heating mode. Conversely, electrical energy—as the primary input for modern cooling systems—is more valuable when the outside temperature is warm, and the indoor space is in cooling mode.

Despite these thermally dependent demands, conventional solar harvesters are restricted to a single continuous mode of sunlight harvesting (3). Solar absorbers—based on metallic plasmonics (4), surface texture (5), and thin-film or composite-based interference effects (6)—are optimized to continuously convert available sunlight into heat. On the other hand, solar photovoltaics—traditionally made of silicon-based semiconductors (7), and more recently of perovskite (8), dye-sensitized (9), organic (10), polymeric (11), and quantum dot (12) materials—are designed to continuously convert available sunlight into electricity. A preferred alternative could passively provide heat when the outside temperature is cool (the indoor space is in heating mode) but provide electricity when the outside temperature is warm (the indoor space is in cooling mode). However, to date, no such self-regulated, dual-mode solar harvester has been realized.

**Fig. 1.**
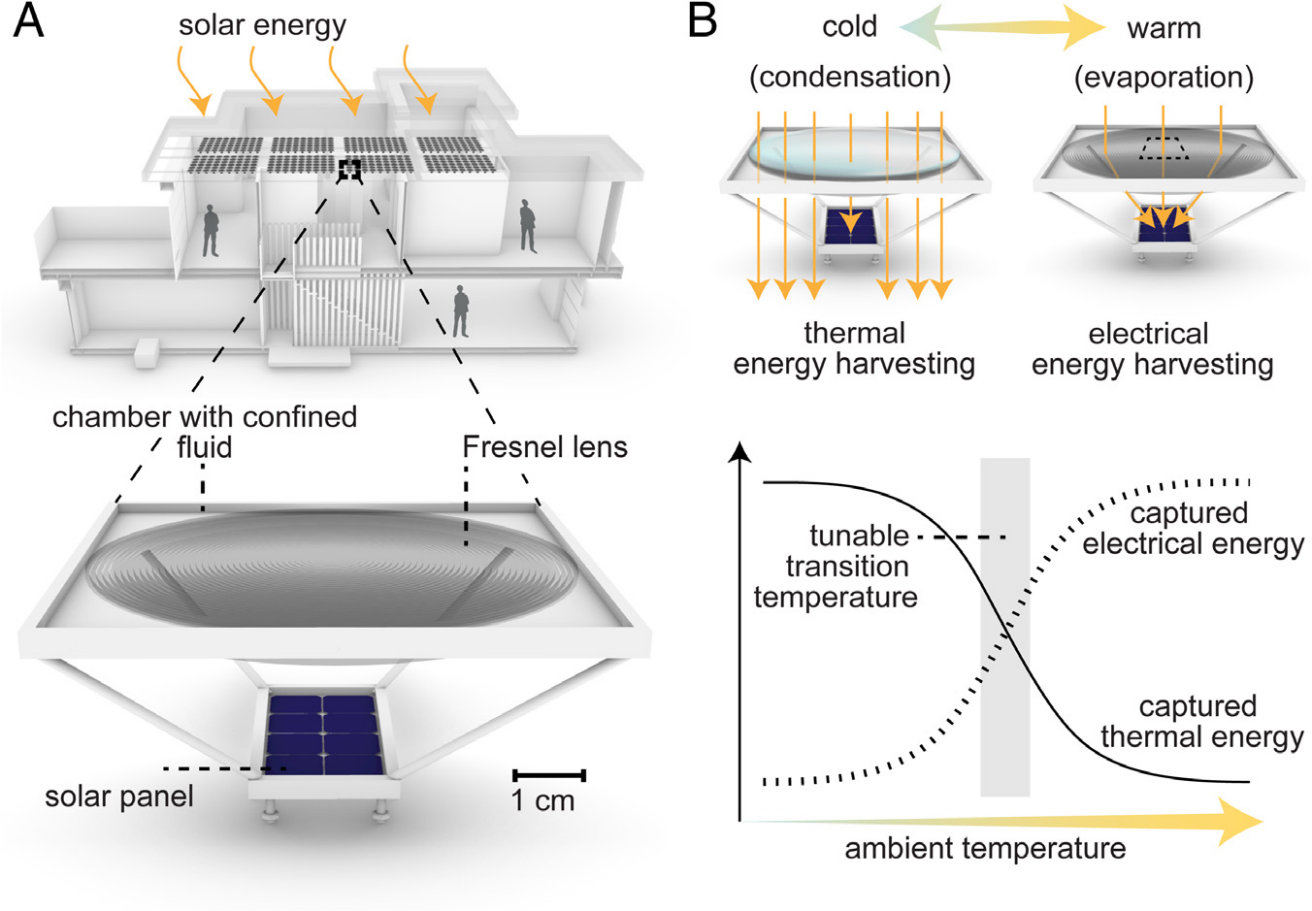
Self-regulated dual-mode solar energy harvesting concept. (*A*) Schematic of exemplary devices which are widely applicable in the built environment, for instance as surfaces atop vehicles, greenhouses, and residential or commercial buildings, and involve only a small number of inexpensive components. (*B*) Working principle. As ambient temperatures change, the devices passively toggle relative yields of thermal and electrical energy through the condensation and evaporation of a fluid layer. The transition temperature separating dominant energy harvesting modes can be tuned by adjusting the fixed amount of fluid confined above the Fresnel lens (FL).

Here, we introduce a concept of self-regulated dual-mode indoor solar energy harvesting widely applicable in the built environment, by treating a liquid layer trapped above a Fresnel lens (FL) as a phase-changing switch within a geometric solar waveguide (Fig. 1*A*). We realized this concept by building passive devices comprising an enclosed layer of water above a microstructured FL concentrator. Devices refract sunlight toward a photovoltaic (PV) cell when the water is in vapor form (greater than the surface’s dew point temperature) but toward an indoor space when in liquid form (less than the surface’s dew point temperature) (Fig. 1*B*). By experimentally simulating environmental heating and cooling cycles, we showed that these harvesters increase indoor solar heating in the cold and PV light exposure in the heat. These results lay the foundation for multimode solar harvesting technology.

## Results

**Fig. 2.**
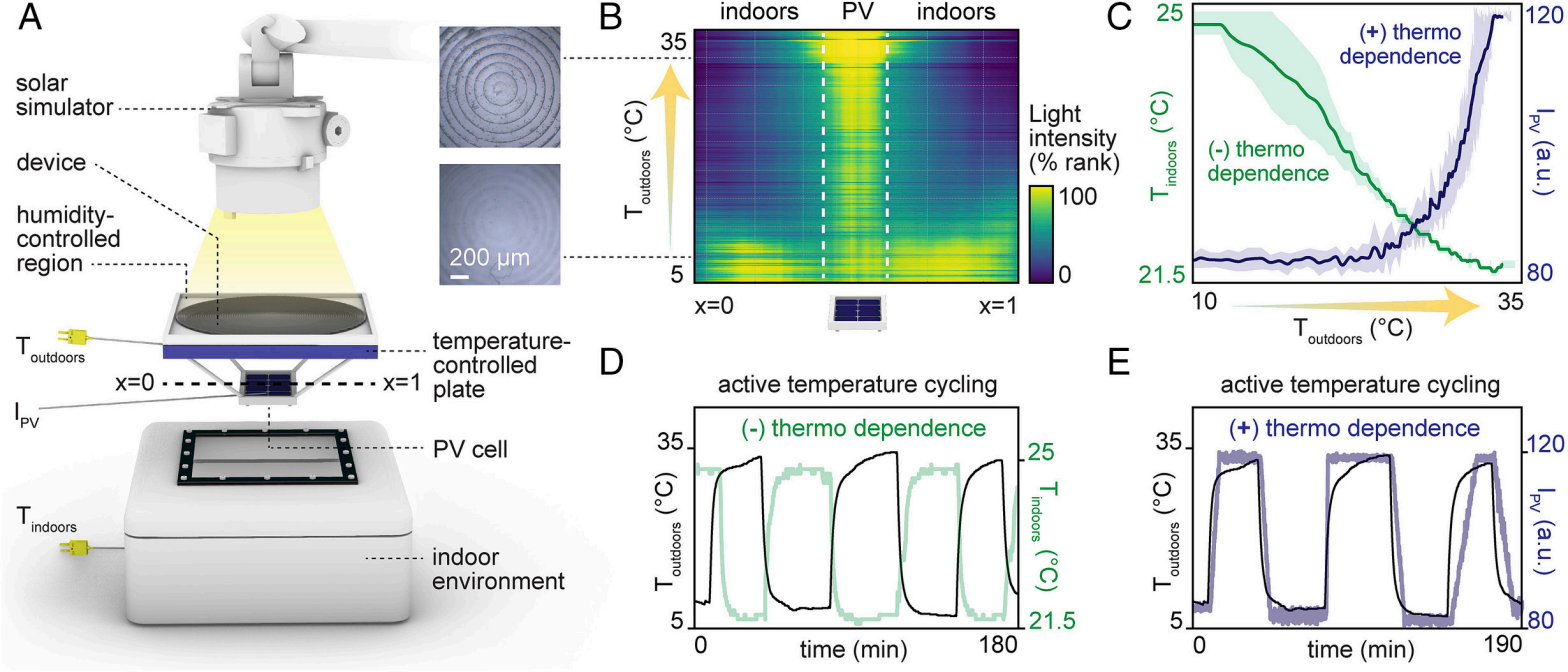
Experimental demonstration of self-regulated dual-mode solar energy harvesting. (*A*) Experimental setup to measure indoor temperatures and PV light intensity as outdoor temperatures change along the FL, where T_outdoors_ is the temperature measured directly beneath the FL, T_indoors_ is the temperature measured along an absorbing surface within the indoor environment, and I_PV_ is the light intensity measured atop the PV cell. (*B*) Light refraction behavior (horizontal axis) changes over time (vertical axis; across a 256 s time interval), as the outdoor temperature is increased from below to above the dew point. Specifically, as the outdoor temperature is experimentally increased from 5 to 35 °C, devices transition to refracting light toward the PV cell and away from the indoor environment. This behavior is shown using relative light intensity measured along a temporary surface slotted across the focal plane (between x = 0 to x = 1). The micrograph insets show the FL both above and below the transition dew point temperature. (*C*) As a result, indoor temperature decreases (negative thermo-dependence), and PV light intensity increases (positive thermo-dependence), with a controlled increase in outdoor temperature. (*D* and *E*) This passive dual-mode self-regulation behavior is observed over repeated experimentally simulated heating/cooling cycles.

Our approach is based on the premise that the effective refractive indices (RIs) between the vapor and liquid phase of a fluid are distinct and that the angle at which light refracts when it passes between two media depends on their RI mismatch. These principles allowed us to develop exemplary passive dual-mode solar harvesters that comprise a microstructured FL, an enclosed layer of water above the FL, and a PV cell with a comparatively small footprint beneath the FL (Fig. 2*A*). The PV cell and indoor environment beneath it serve as two possible energy sinks for refracted sunlight. The phase-changing water layer serves to mediate the refractive path of that sunlight, toggling as a function of temperature the ultimate destination to which solar energy is delivered.

We designed our harvesters based on three criteria: 1) the FL must refract sunlight onto a focal region the size of the PV when the fluid above the lens is in vapor phase; 2) the FL must refract sunlight onto a focal region much bigger than the PV (such that the majority of solar energy bypasses the PV and is instead transmitted into an indoor space below) when the fluid above the lens is in liquid phase; and 3) the phase transition temperature must be calibrated to the desirable shift in need between electricity and heat.

To satisfy the first two requirements, we made the FL out of a polymeric acrylic resin with a RI (~1.38) that closely matches that of liquid water (~1.33) placed above it, but that differs substantially from humid air (~1, assuming a relative humidity (RH) of 65%, room air temperature of 21 °C, atmospheric pressure, and standard CO_2_ levels) (13) (a photograph of our functional device is displayed in *SI Appendix*, Fig. S1). To satisfy the third requirement, we fixed the RH of the enclosed space above the FL to 65%, corresponding to a dew point temperature of ~15 °C (assuming an air temperature of 21 °C). Because the transition temperature between modes of energy harvesting is simply this dew point, it can be easily changed by adjusting the RH. Such tunability may be important for indoor environments that have variable heating and electricity demands over times of day, seasons, and occupancy modes, and for which there may be a desire to bias production of one or the other energy form at key time intervals.

When we experimentally produced both warm (above the dew point) and cool (below the dew point) outdoor temperatures (T_outdoors_) surrounding the harvester (experimental setup illustrated in Fig. 2*A*; temperature control details in Materials and Methods), we observed a clear change in focusing behavior (measured as optical intensity across the focal plane) corresponding to the evaporation and condensation of water above the FL (Fig. 2*B*). At steady state, no liquid water was observed atop the FL for T_outdoors_ > 16 °C, and sunlight was concentrated primarily along the PV surface. For T_outdoors_ < 14 °C, however, a stable liquid film (1.8 ± 0.15 mm thick) consistently formed, allowing much of the sunlight to bypass the PV cell indoors.

Sunlight is absorbed as heat when transmitted indoors. Accordingly, while the device increases solar focusing on a PV above the dew point temperature, it increases indoor solar heating when below the dew point temperature. As demonstrated in Fig. 2*C*, when the simulated outdoor temperature was increased from 10 to 35 °C, the indoor temperature decreased from ~25 °C (excessive) to ~21.5 °C (comfortable), while the relative light intensity on the PV increased by ~50% (from ~80 to ~120 a.u.). These opposite negative and positive thermo-dependent trends are repeatable over multiple cycles, as shown in Fig. 2 *D* and *E*, respectively. The general nature of these results is further supported by supplementary data in the *SI Appendix* which show that: 1) for all steady state T_outdoors_ > 16 °C studied (17, 20, 25 and 30 °C), I_PV_ and T_indoors_ values converged to an equilibrium I_PV_-biased state (*SI Appendix*, Figs. S2–S4 and *Supplementary Note* 1); 2) for steady state T_outdoors_ < 14 °C studied (11, 13 °C), I_PV_ and T_indoors_ values converged to a distinct equilibrium T_indoors_-biased state (*SI Appendix*, Figs. S2–S4 and *Supplementary Note* 1); 3) times to equilibrium varied with T_outdoors_, likely due to changing evaporation or condensation rates (*SI Appendix*, Fig. S3); 4) within a transition threshold (13 °C < T_outdoors_ < 17 °C), no equilibrium states were reached (*SI Appendix*, Fig. S2); 5) consistent temperature-dependent behavior is observed for both low (300 W/m^2^) and high (1,000 W/m^2^) solar irradiance (*SI Appendix*, Fig. S5 and *Supplementary Note* 2); 6) while arbitrary units of light intensity are reported in Fig. 2, equivalent experiments measuring electrical current output from a commercially available PV cell demonstrate the same trends (*SI Appendix*, Fig. S6 and *Supplementary Note* 3).

We also modeled and demonstrated the ranges for stable device performance and performance degradation under different angles of solar incidence (0, 15, and 45°) (*SI Appendix*, Figs. S7–S10 and *Supplementary Note* 4) and module tilting (0, 15, and 45°) (*SI Appendix*, Figs. S11–S13 and *Supplementary Note* 5). These measurements allowed us to find the hours of the day and year for which performance is optimal, given specified geographies and module orientations (*SI Appendix*, Fig. S14 and *Supplementary Note* 6).

## Discussion

So long as the established design criteria are satisfied, these observed effects should be general across different FL materials including polymers and glasses, which can be shaped using various scalable and commercially available batch or roll-to-roll processing techniques (e.g., thermal imprinting of thermoplastic polymers, photocuring of light-curable resins, and molding polymers or solvated ceramics on templates) (14–17), and different fluids (e.g., organic solvents) (experimental data for additional materials combinations are presented in *SI Appendix*, Fig. S15 and *Supplementary Note* 7). Moreover, our concept can be generalized to include further energy harvesting modes (for example, by replacing the PV cell with a water flow pipe for domestic water heating). Otherwise, the system might be adapted to include active modes of control—for example, involving the injection and exchange of liquids of different RIs into, or the active regulation of RH level within, the cavity above the FL, or by using adaptive FL materials that autonomously change the FL geometry and thus optical properties in response to temperature. Overall, our work provides the design criteria and experimental proof-of-concept for dual-mode solar harvesting technologies. Future studies should address issues surrounding FL geometry, surface chemistry optimization, and moisture sealing.

## Materials and Methods

The acrylic resin FL (NOA 1348, Norland Products) was cast from a commercially available Polymethylmethacrylate (PMMA) mold purchased from Edmund Optics with a facet height of ~0.15 mm, pitch of ~0.2 mm, and a concentration factor of 10 (5 cm FL, 5 mm focal spot along the focal plane). The stand-in polycrystalline silicon PV cell used is also commercially available from Uxcell. The device frame—connecting the FL to the PV cell—was printed using a Prusa MK4S three-dimensional printer from Polylactic Acid filament material. Outdoor temperatures (along the FL) were experimentally simulated by cycling precooled or preheated water (by a chiller or hot plate, respectively) within a transparent, PMMA water-flow cavity directly beneath the FL. Indoor and outdoor temperatures were measured using K-type thermocouples. The thermocouple placed indoors was taped to an interior absorbing surface (Welstik black tape) with an average solar absorptivity between 0.3 and 2.5 μm of 94%, as measured using a Cary 7000 spectrophotometer (Agilent) with an integrating sphere accessory (DRA-2500, Labsphere). The indoor environment was made from Styrofoam walls (3.2 cm thick), had dimensions of 15.2 × 12.7 × 16.5 cm, and featured a double-paned skylight—through which sunlight could enter the indoor environment below—made from two 0.05-mm-thick Polyethylene sheets (McMaster-Carr) separated by a 3 cm air gap. The interior absorbing surface on which indoor temperature was measured was placed 5.5 cm beneath the FL focal point and 1.5 cm beneath the bottom pane of the skylight indoors. Fixed relative humidity above the FL was experimentally simulated using an ultrasonic humidifier (Ledonti) and measured using a relative humidity sensor (TSI, 9565-P). The humidity in the laboratory where experiments were conducted was measured at ~25% with the same sensor, although measured experimental results are expected to be independent of laboratory humidity level. The thicknesses of liquid water films above the FL were measured with a digital caliper (PE tools) at multiple positions. Relative spatial light intensity—as depicted in Fig. 2*B*—was measured by taking pixel values along a temporary surface slotted across the focal plane (between x = 0 to x = 1), photographed using a digital camera (Canon, EOS Rebel T3i) and analyzed using a custom script developed in Python. Relative spatial light intensity along the PV cell—as depicted in Fig. 2 *C* and *E*—was measured by taking the average pixel value from a 0.25 cm^2^ region at the center of the PV cell, photographed using the same digital camera and analyzed using a similar Python script. Sunlight was simulated using a Lishio EKE 21 V 50 W bulb and Fiber-lite M I-152 high intensity illuminator, providing 300 or 1,000 W/m^2^ of solar irradiance, measured along the FL using a pyranometer (SP-510-SS, Apogee). All experiments were conducted under 300 W/m^2^ solar irradiance, except for those explicitly conducted to study the effects of high solar irradiance (1,000 W/m^2^), described in *SI Appendix*, Fig. S5 and *Supplementary Note* 2. The spatial light intensity distribution across the FL is detailed in *SI Appendix*, Figs. S16 and S17 and *Supplementary Note* 8.

## Supplementary Material

Appendix 01 (PDF)

## Data Availability

Study data are included in the article and/or *SI Appendix*.

## References

[1] International Energy Agency, Global Energy & CO2 Status Report 2017 (IEA, Paris, France, 2018). https://www.iea.org/reports/global-energy-co2-status-report-2017.

[2] U.S. Department of Energy, Quadrennial Technology Review: An Assessment of Energy Technologies and Research Opportunities (United States Department of Energy, 2015), Chap. 5.

[3] C. G. Granqvist, Solar energy materials. Adv. Mater. **15**, 1789–1803 (2003), 10.1002/adma.200300378.

[4] M. Chen, Y. He, Plasmonic nanostructures for broadband solar absorption based on the intrinsic absorption of metals. Sol. Energy Mater. Sol. Cells **188**, 156–163 (2018), 10.1016/j.solmat.2018.09.003.

[5] X.-H. Gao, Z.-M. Guo, Q.-F. Geng, P.-J. Ma, G. Liu, Enhanced absorptance of surface-textured tungsten thin film for solar absorber. Surf. Eng. **32**, 840–845 (2016), 10.1080/02670844.2016.1187466.

[6] J. Zhang , Solar selective absorber for emerging sustainable applications. Adv. Energy Sustain. Res. **3**, 2100195 (2022), 10.1002/aesr.202100195.

[7] R. A. Marques Lameirinhas, J. P. N. Torres, J. P. de Melo Cunha, A photovoltaic technology review: History, fundamentals and applications. Energies **15**, 1823 (2022).

[8] H. S. Jung, N. G. Park, Perovskite solar cells: From materials to devices. Small **11**, 10–25 (2015). 25358818 10.1002/smll.201402767

[9] A. Hagfeldt, G. Boschloo, L. Sun, L. Kloo, H. Pettersson, Dye-sensitized solar cells. Chem. Rev. **110**, 6595–6663 (2010). 20831177 10.1021/cr900356p

[10] H. Hoppe, N. S. Sariciftci, Organic solar cells: An overview. J. Mater. Res. **19**, 1924–1945 (2004).

[11] K. M. Coakley, M. D. McGehee, Conjugated polymer photovoltaic cells. Chem. Mater. **16**, 4533–4542 (2004).

[12] A. J. Nozik, Quantum dot solar cells. Phys. E Low-Dimens. Syst. Nanostruct. **14**, 115–120 (2002).

[13] L. Dettwiller, Short review on the refractive index of air as a function of temperature, pressure, humidity and ionization. arXiv [Preprint] (2022). https://arxiv.org/abs/2204.02603 (Accessed 15 October 2025).

[14] X. Zhang, K. Liu, X. Shan, Y. Liu, Roll-to-roll embossing of optical linear Fresnel lens polymer film for solar concentration. Opt. Express **22**, A1835–A1842 (2014), 10.1364/OE.22.0A1835. 25607497

[15] Y. Tripanagnostopoulos, C. Siabekou, J. K. Tonui, The Fresnel lens concept for solar control of buildings. Solar Energy **81**, 661–675 (2007), 10.1016/j.solener.2006.08.013.

[16] D. Sato, N. Yamada, Design and testing of highly transparent concentrator photovoltaic modules for efficient dual-land-use applications. Energy Sci. Eng. **8**, 779–788 (2020), 10.1002/ese3.550.

[17] G. Nardin , Industrialization of hybrid Si/III–V and translucent planar micro-tracking modules. Prog. Photovoltaics Res. Appl. **29**, 819–834 (2021), 10.1002/pip.3387.

