# Supplementary material for: Self-regulated dual-mode solar energy harvesting
Source: Proc Natl Acad Sci U S A. 2026 Mar 24;123(13):e2534717123. doi: 10.1073/pnas.2534717123 (PMC13037869; doi:10.1073/pnas.2534717123)
Supplement: Supplementary file 1 — Appendix 01 (PDF) [file pnas.2534717123.sapp.pdf]

PNAS

Supplemental Information file for

# Self-regulated dual-mode solar energy harvesting

Raphael Kay[a]*, Rafiq Omair[a], Joanna Aizenberg[a,b]*

[a]Harvard John A. Paulson School of Engineering and Applied Sciences, Harvard University, Cambridge, MA 02138, USA.
[b]Department of Chemistry and Chemical Biology, Harvard University, Cambridge, MA 02138, USA.

*Correspondence to: Raphael Kay, Joanna Aizenberg

**Email:** rkay@seas.harvard.edu, jaiz@seas.harvard.edu

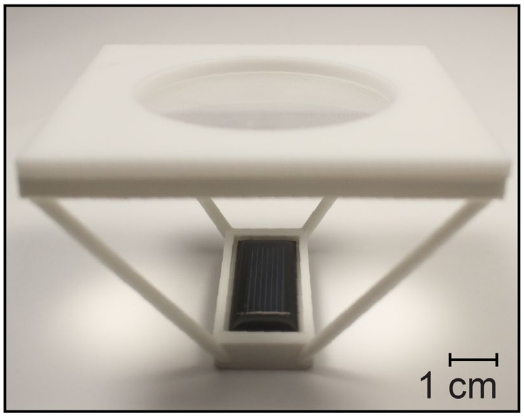


**Figure S1. Device photograph.** Details of the device design and manufacture are included in the Materials and Methods section.

## Supplementary Note 1: Device performance near the dew point.

We investigated the behavior of our system around the dew point temperature (~15 °C). We first cooled the Fresnel lens (FL) to reach a stable below-dew-point temperature (~10 °C), before heating it to various temperatures ($T_{outdoors}$, as defined in Fig. 2a) below the dew point (11, 13 °C), above the dew point (17 °C, 20 °C, 25 °C, 30 °C), and right around the dew point (15 °C, 16 °C). For steady state $T_{outdoors}$ > 16 °C, we never observed liquid water atop the FL. For steady state $T_{outdoors}$ < 14 °C, a stable liquid film (1.8 +/- 0.15 mm thick) consistently formed. Accordingly, $I_{PV}$ consistently converged to an equilibrium minimum ($I_{PV}$ ~85 a.u.) for $T_{outdoors}$ < 14 °C, and an equilibrium maximum ($I_{PV}$ ~120 a.u.) for $T_{outdoors}$ > 16 °C (**Figure S2**). Within a transition threshold right around the dew point (13 °C < $T_{outdoors}$ < 17 °C), steady state $I_{PV}$ values were measured in between the two equilibrium states ($I_{PV}$ ~90-100 a.u.) (**Figure S2**).

In addition, we ran experiments to study transition timescales, by heating the device from below the dew point (~10 °C) to different temperatures above the dew point (**Figure S3**). We found that while the final equilibrium state was approximately the same for all experiments (devices heated to 17 °C, 20 °C, 25 °C, 30 °C), the time to reach this equilibrium state varied, as evaporation rates depend on Fresnel lens temperature.

Experiments showing natural variability in transition dynamics across outdoor temperature ($T_{outdoors}$) cycles are presented in **Figure S4**.

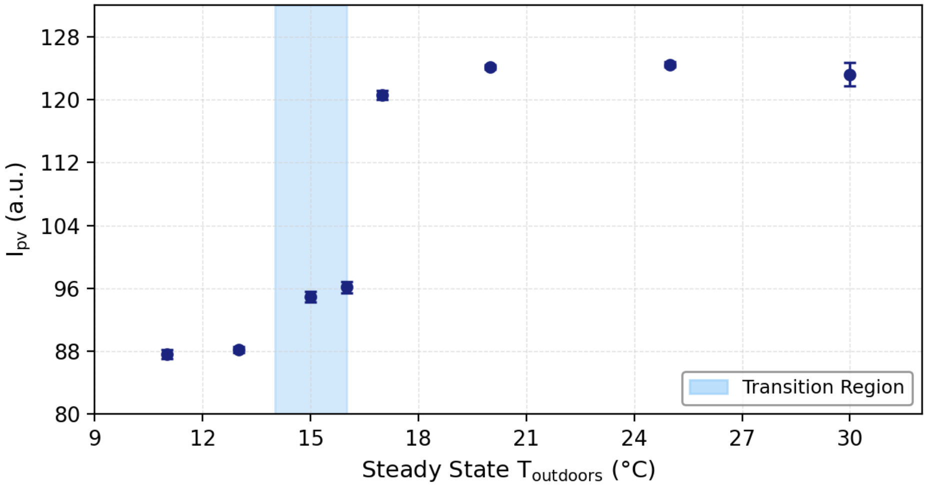


**Figure S2. Device performance near the dew point.** Measured $I_{PV}$ values at steady state outdoor temperatures below the dew point (11, 13 °C) correspond to characteristic equilibrium thermal harvesting mode performance ($I_{PV}$ ~85 a.u.), while measured $I_{PV}$ values at steady state outdoor temperatures above the dew point (17 °C, 20 °C, 25 °C, 30 °C) correspond to characteristic equilibrium electricity harvesting mode performance ($I_{PV}$ ~120 a.u.). A transition threshold is observed and defined for steady state outdoor temperature between 14-16 °C. The $I_{PV}$ measurements correspond to average values measured over three trials at each temperature, while the error bars correspond to minimum and maximum $I_{PV}$ values.

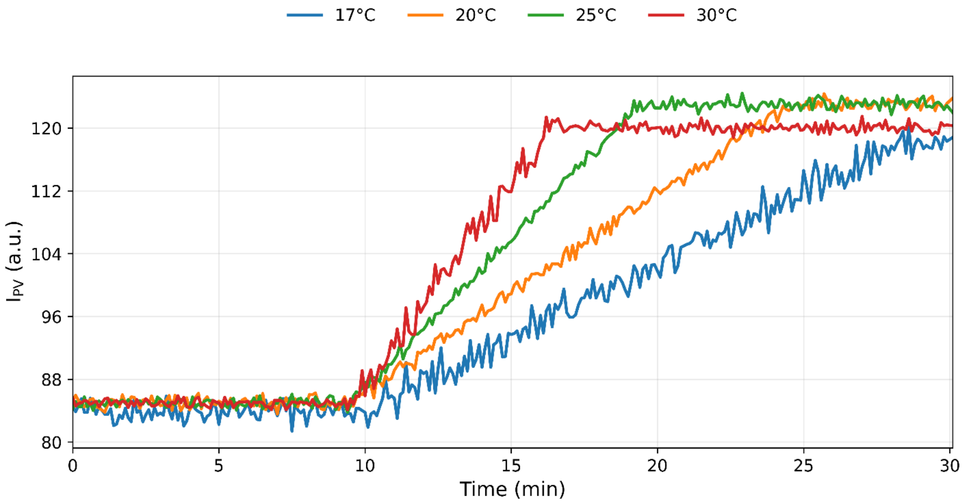


**Figure S3. During state switching, outdoor temperature affects time to equilibrium but not equilibrium.** Measured $I_{PV}$ values over time for experiments involving the controlled increase in outdoor temperature from below the dew point (~10 °C) to various steady state temperatures above the dew point (17 °C, 20 °C, 25 °C, 30 °C). While the final equilibrium $I_{PV}$ values reach approximately similar equilibrium levels, the time to equilibrium depends on $T_{outdoors}$.

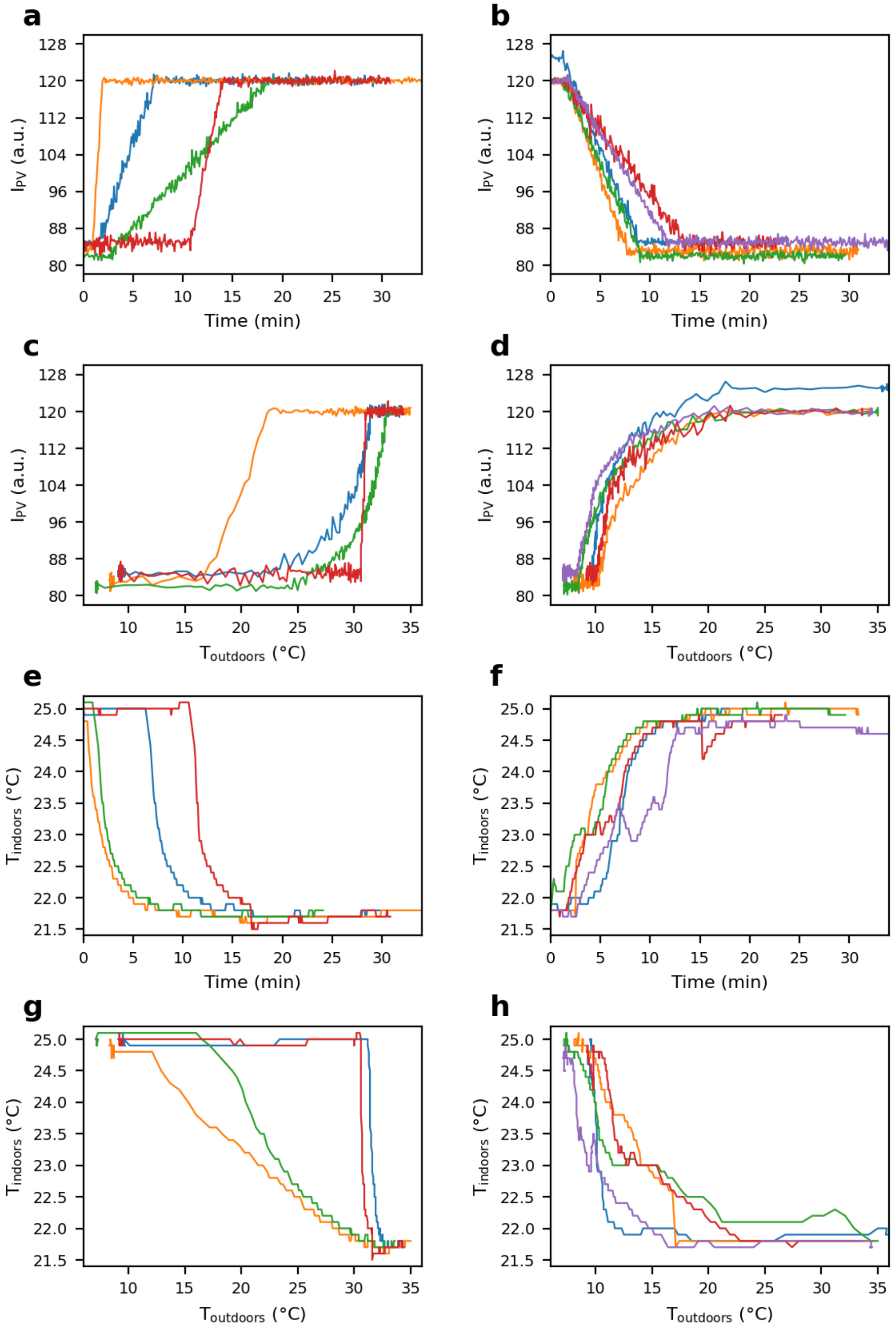


**Figure S4. Natural variability in transition dynamics across outdoor temperature ($T_{outdoors}$) cycles.** (**a-b**) $I_{PV}$ versus time for various (a) heating and (b) cooling temperature cycles, where *t* = 0 is defined as the beginning of each heating or cooling cycle. (**c-d**) $I_{PV}$ versus $T_{outdoors}$ for various (c) heating and (d) cooling cycles. (**e-f**) $T_{indoors}$ versus time for various (e) heating and (f) cooling cycles (same definition of *t* = 0). (**g-h**) $T_{indoors}$ versus $T_{outdoors}$ for various (g) heating and (h) cooling cycles.

**Supplementary Note 2: 1000 W/m$^2$ solar irradiance measurements.**

**Figure S5** shows experimental results that demonstrate similar performance trends as shown in Figure 2, but with solar irradiance of ~1000 W/m$^2$ instead of ~300 W/m$^2$.

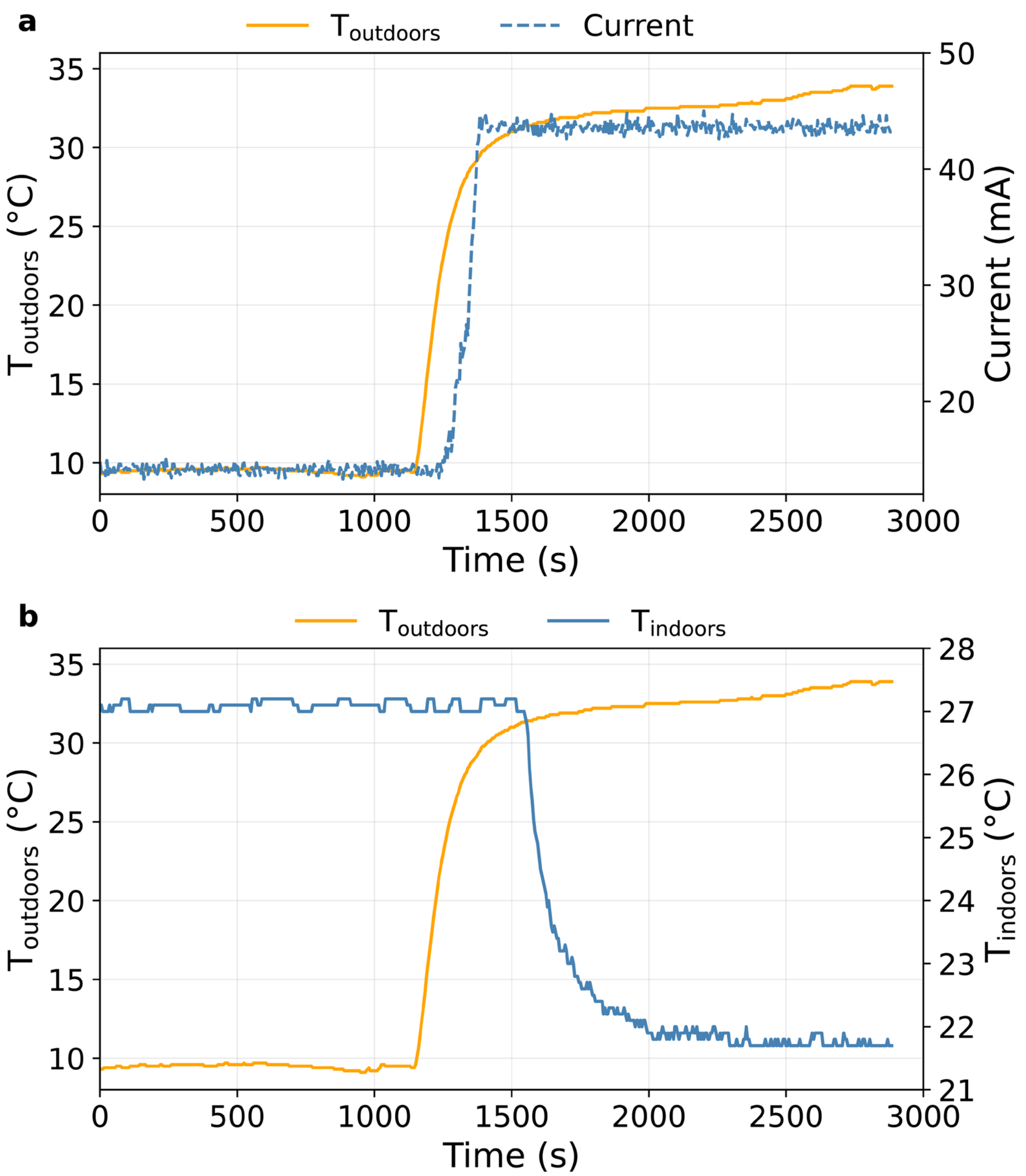


**Figure S5. Device behaviors remain consistent under standard one-sun irradiance conditions (1000 W/m²).** (**a**) PV cell light intensity ($I_{pv}$) changing with controlled $T_{outdoors}$ increase, both plotted over time. (**b**) $T_{indoors}$ changing with controlled $T_{outdoors}$ increase, both plotted over time. Our system exhibits similar temperature dependent performance trends under both 300 W/m$^2$ and 1000 W/m$^2$ simulated solar irradiance.

## Supplementary Note 3: Electrical current measurements.

**Figure S6** shows experimental results that demonstrate similar performance trends as shown in Figure 2, but with measured electrical current from PV cells.

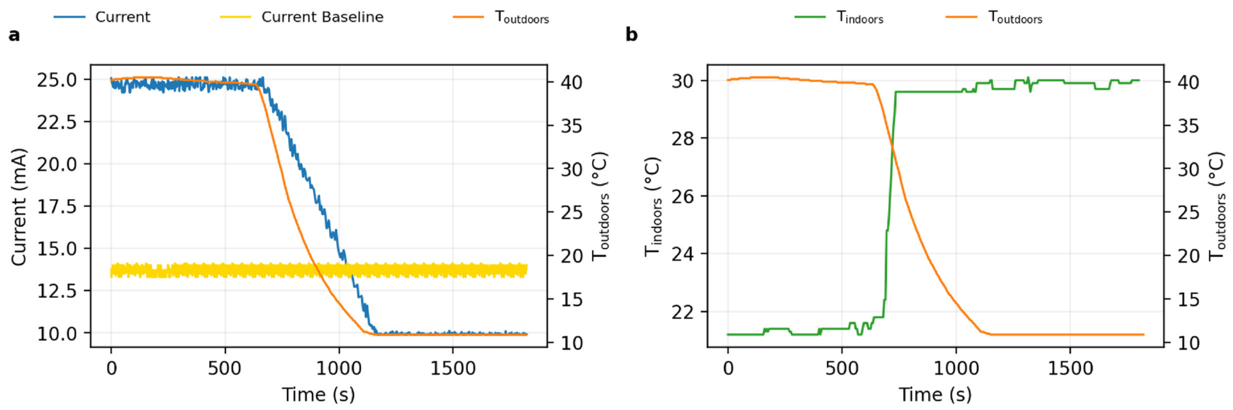


**Figure S6. Measurement of electrical current generation during thermally driven mode switching** (**a**) Photovoltaic output current (mA) changing with controlled $T_{outdoors}$ decrease, both plotted over time. Also plotted is a baseline current reading under simulated sunlight with no Fresnel lens. (**b**) $T_{indoors}$ changing with controlled $T_{outdoors}$ decrease, both plotted over time. The experimental set up used to collect this data can be found in Figure 2a and is further discussed in the Materials and Methods section. An Arduino Uno R3 was connected to the PV cell to collect current.

## Supplementary Note 4: Incidence-angle dependence of measured $I_{PV}$.

The non-zero areal footprint of the PV panel allows for a range of solar incidence angles for which the device maintains its expected evaporation-induced solar concentration behavior. In our case ($W_{PV}$ = 20 mm), we observe consistent solar concentration performance for solar incidence angles up to 15° (**Figure S7**).

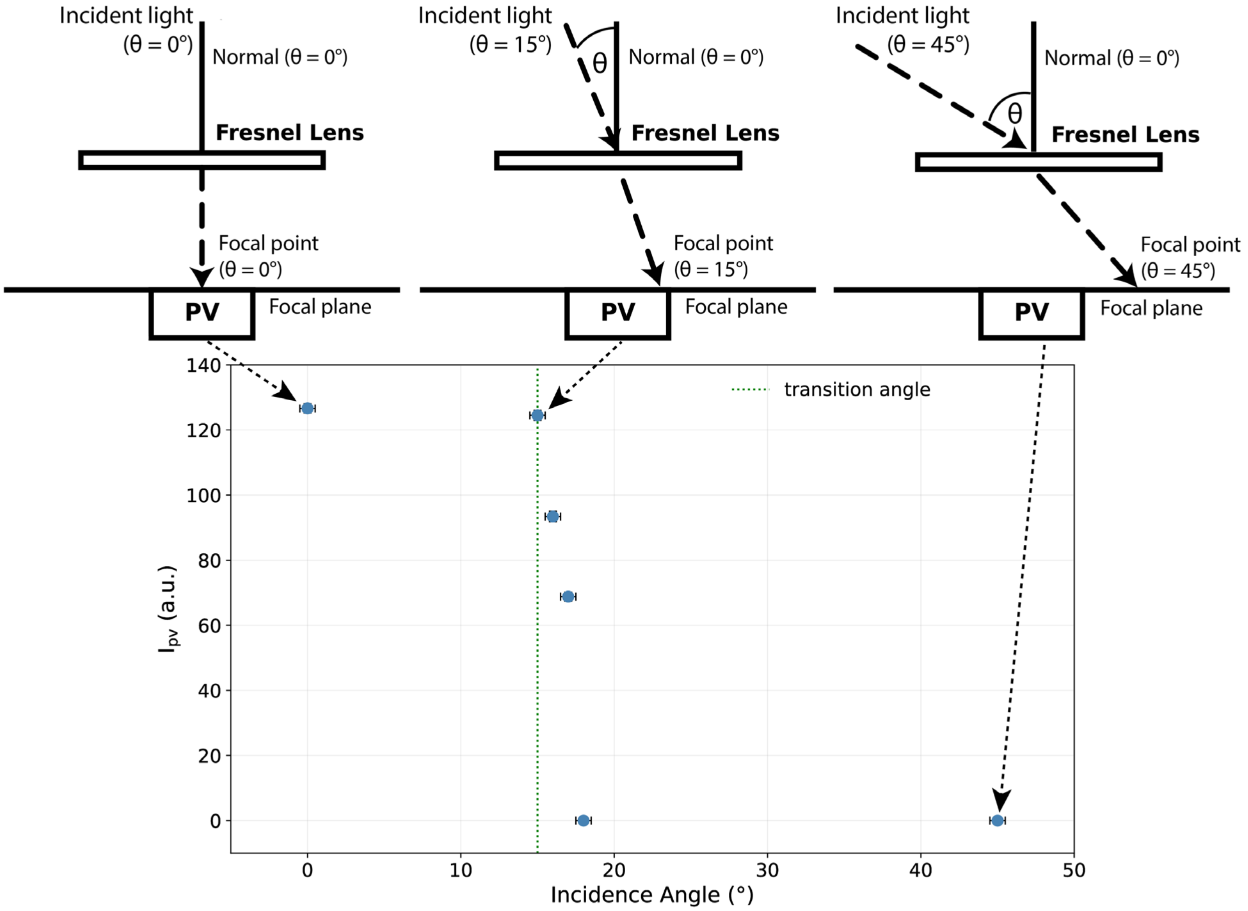


**Figure S7. Peak $I_{PV}$ versus angle of incidence (when $T_{outdoors}$ = 35°C), showing a robustness threshold for which PV signal does not depend on the incoming θ.** Top: Representative sketches corresponding to three distinct angles of incidence scenarios (θ = 0°, 15°, 45°, from left to right) illustrating various focal spot displacements. For θ ≤ 15°, the focal spot is fully captured by the PV active area. Once θ > 15°, the spot crosses the PV edge and becomes partially and eventually fully uncaptured, causing a step-like reduction in measured $I_{PV}$. The $I_{PV}$ measurements correspond to average values measured over three trials at each incidence angle, while the error bars correspond to minimum and maximum measured $I_{PV}$ values, as well as experimental measurement error.

This angular robustness can be explained geometrically (**Figure S8**).

In general, a collimated solar beam incident on a FL at angle θ produces a focused spot on the focal plane. For non-zero θ, and allowing $\theta = \theta_R$, a ray shift is set by the right-triangle formed by the focal length, f, and the lateral walk-off, x, where:

(Eq. 1) $$x = f\tan(\theta)$$

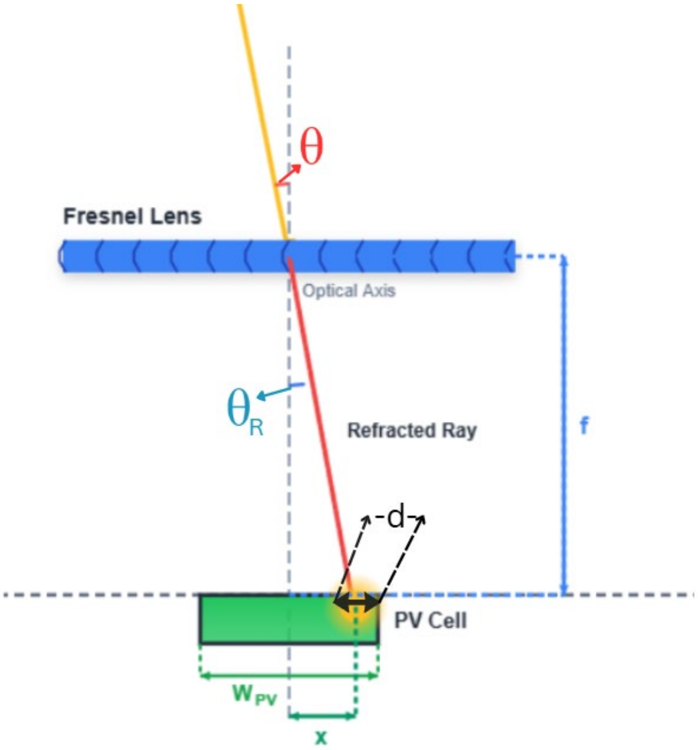


**Figure S8. Geometric picture to explain device performance under varying angles of incidence (θ).**

Here, $\theta = \theta_R$ is assumed to be true in the dry device state, because of a symmetrical index mismatch below and above the FL. The PV is centered on the optical axis and spans a width ($W_{PV}$) on the focal plane. The spot has diameter d, and the spot center remains on the PV provided:

(Eq. 2) $$|x| \leq \frac{W_{PV}}{2}$$

Combining Eq. (1-2) yields the maximum angle for which the spot center stays on the PV:

(Eq. 3) $$\theta_{center,max} = \arctan\left(\frac{\left(\frac{W_{PV}}{2}\right)}{f}\right)$$

For our demonstrated system (f = 38.10 mm, $W_{PV}$ = 20 mm, d = 5 mm):

$$\theta_{center,max} = \arctan\left(\frac{10}{38.10}\right) = \sim 15°$$

These modeled results agree well with experimental results that show a fall in $I_{PV}$ performance for solar incidence angles greater than 15° (**Figure S7**).

These results held across multiple heating and cooling cycles (**Figures S9-10**).

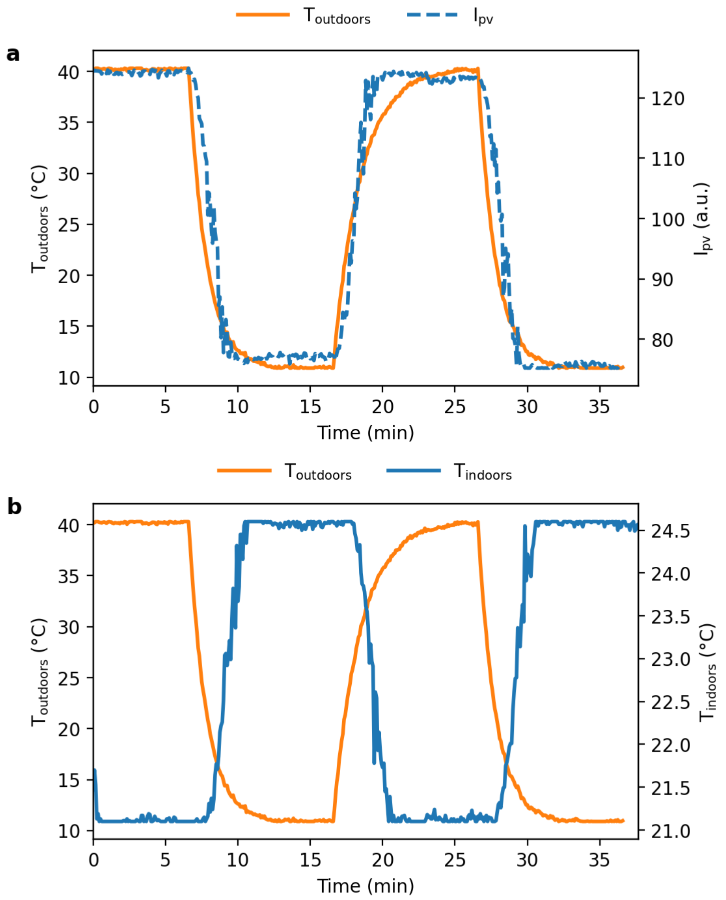


**Figure S9. Effect of illumination at 15° angle of solar incidence (~220 W/m²) on measured thermally driven switching behavior.** (**a**) $I_{PV}$ (defined in Fig. 2a) moves in phase with controlled cyclic changes to outdoor temperature over time. (**b**) Indoor temperature ($T_{indoors}$, defined in Fig. 2a) moves out of phase with controlled cyclic changes to outdoor temperature over time. The experimental set up used to collect this data can be found in Figure 2a and is discussed further in the Materials and Methods section.

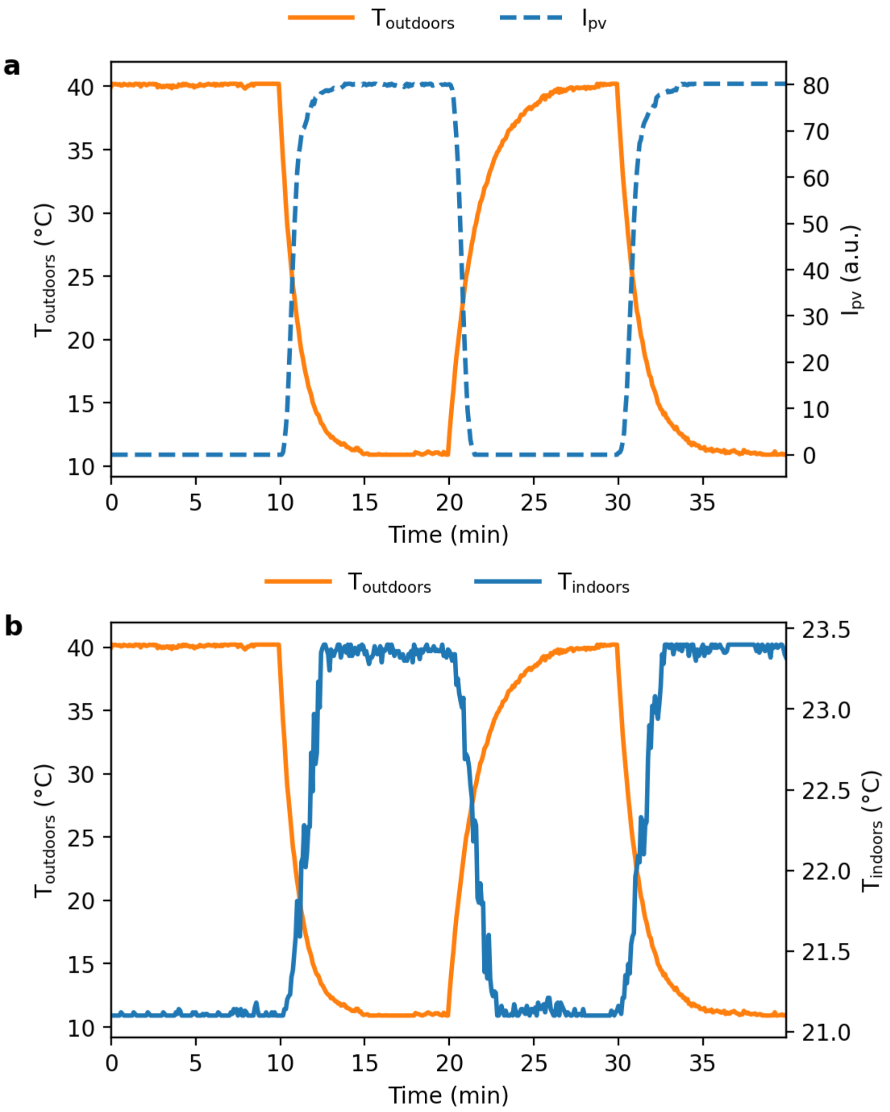


**Figure S10. Effect of illumination at 45° angle of incidence (~140 W/m²) on measured thermally driven switching behavior.** (**a**) $I_{PV}$ (defined in Fig. 2a) moves out of phase with controlled cyclic changes to outdoor temperature over time. (**b**) Indoor temperature ($T_{indoors}$, defined in Fig. 2a) moves out of phase with controlled cyclic changes to outdoor temperature over time. The experimental set up used to collect this data can be found in Figure 2a and is discussed further in the Materials and Methods section.

## Supplementary Note 5: Module tilt-angle robustness.

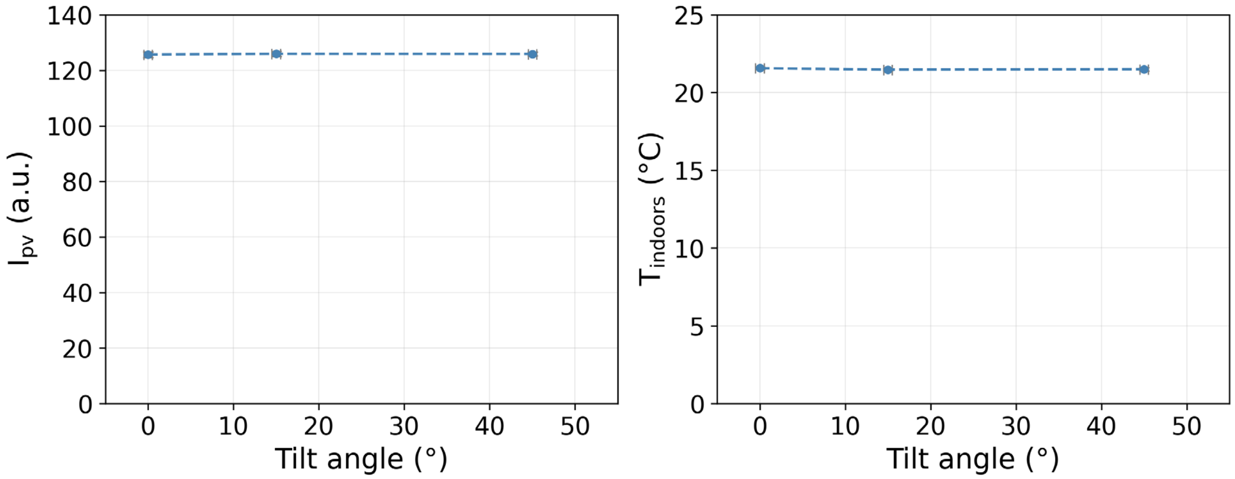


**Figure S11. Device performance robustness to module tilting.** Steady state $I_{PV}$ (left) and steady state $T_{indoors}$ (right) for experiments conducted with incident light normal to the device at different module tilt angles. The $I_{PV}$ and $T_{indoors}$ measurements correspond to average values measured over three trials at each tilt angle, while the error bars correspond to minimum and maximum $I_{PV}$ and $T_{indoors}$ values, as well as experimental measurement error.

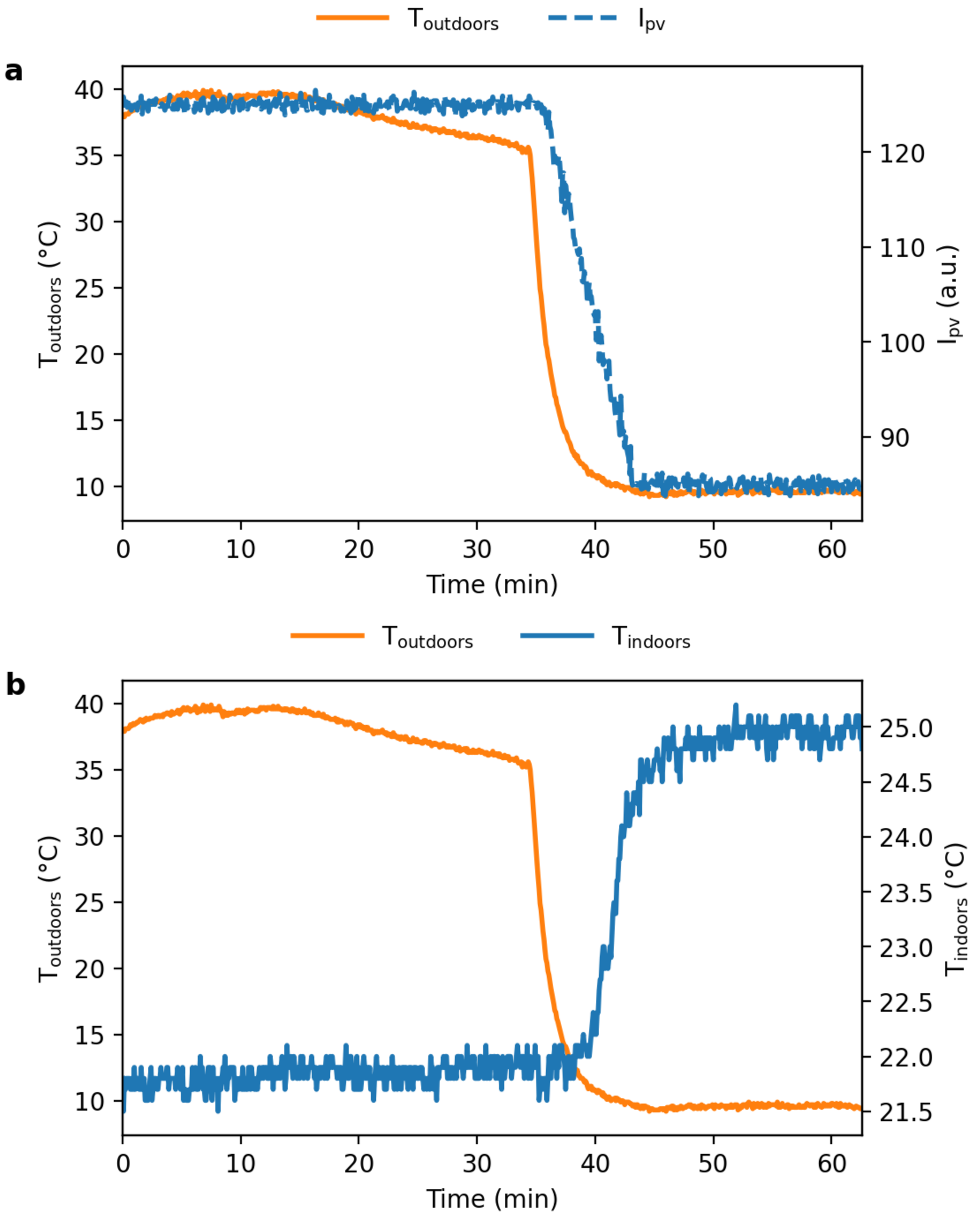


**Figure S12. Device performance remains constant at module tilt angles of 15°.** Time traces of (**a**) $I_{PV}$ and (**b**) $T_{indoors}$ measured during an imposed temperature cycle ($T_{outdoors}$). The characteristic dual-mode response is maintained under 15° tilt.

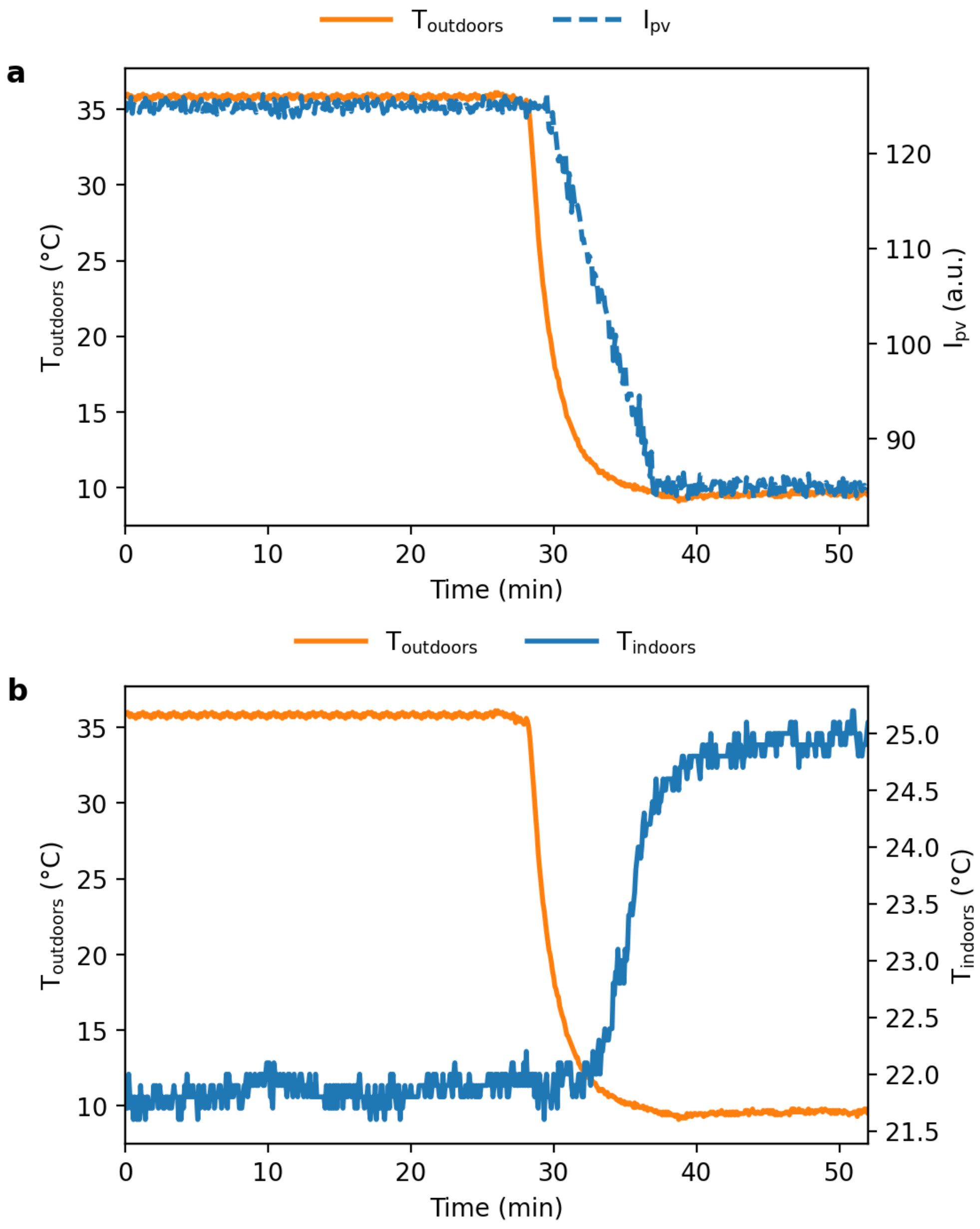


**Figure S13. Device performance remains constant at module tilt angles of 45°.** Time traces of (**a**) $I_{PV}$ and (**b**) $T_{indoors}$ measured during an imposed temperature cycle ($T_{outdoors}$). The characteristic dual-mode response is maintained under 45° tilt.

**Supplementary Note 6: Time of year operation simulations.**

By using solar irradiance data obtained from open-source weather files (for Boston, MA, United States; TMYx.2004-2018 climate data), we found the hours of the year for which solar irradiation is concentrated onto our PV panel (assuming a 20° tolerance to off-normal irradiance, based on reasonable angle of incidence assumptions from **Eq. 1-3**). These hours are visualized in **Figure S14a-c**, for fixed device altitude tilts of 20°, 30°, and 40°, respectively. In general, concentration performance is steady during peak cooling hours (midday, summer), when electricity demand is highest, especially for tilt altitudes of 20°, 30°.

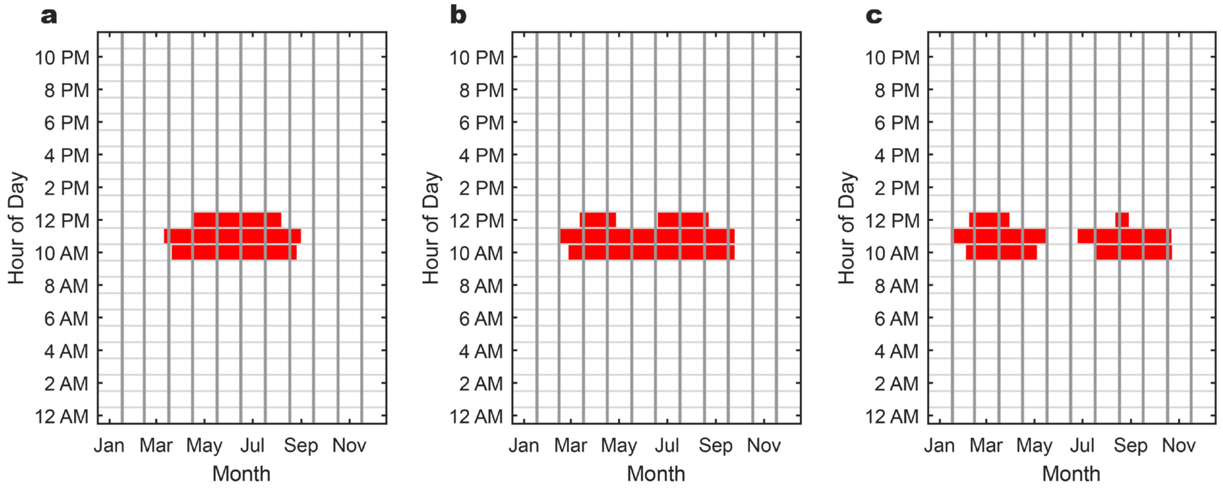


**Figure S14. Hours of the year for which sunlight is concentrated onto our PV panel (visualized in red).** We assume a 20° tolerance to off-normal irradiance, based on angle of incidence robustness assumptions from **Figure S7-8** and **Eq. 1-3**. Data corresponds to device altitude tilts of (**a**) 20°, (**b**) 30°, and (**c**) 40°, respectively. Solar irradiance data is gathered from open-source weather files (for Boston, MA, United States; TMYx.2004-2018 climate data). We graciously thank Prof. Alstan Jakubiec (University of Toronto) for simulating data and developing these plots.

**Supplementary Note 7: Generality of the Fresnel waveguiding principle across different fluid-solid pairs.**

We performed experiments involving active flows of liquids to and from the cavity above the Fresnel lens – based on two distinct polymer lens/fluid pair selections (e.g., polymethylmethacrylate (PMMA (acrylic), RI ~1.49) and glycerol (RI ~1.48) and polydimethylsiloxane (PDMS, RI ~1.42) and silicone oil (RI ~1.42)), to show expanded possibilities for dual-mode solar harvesting performance (**Figure S15**).

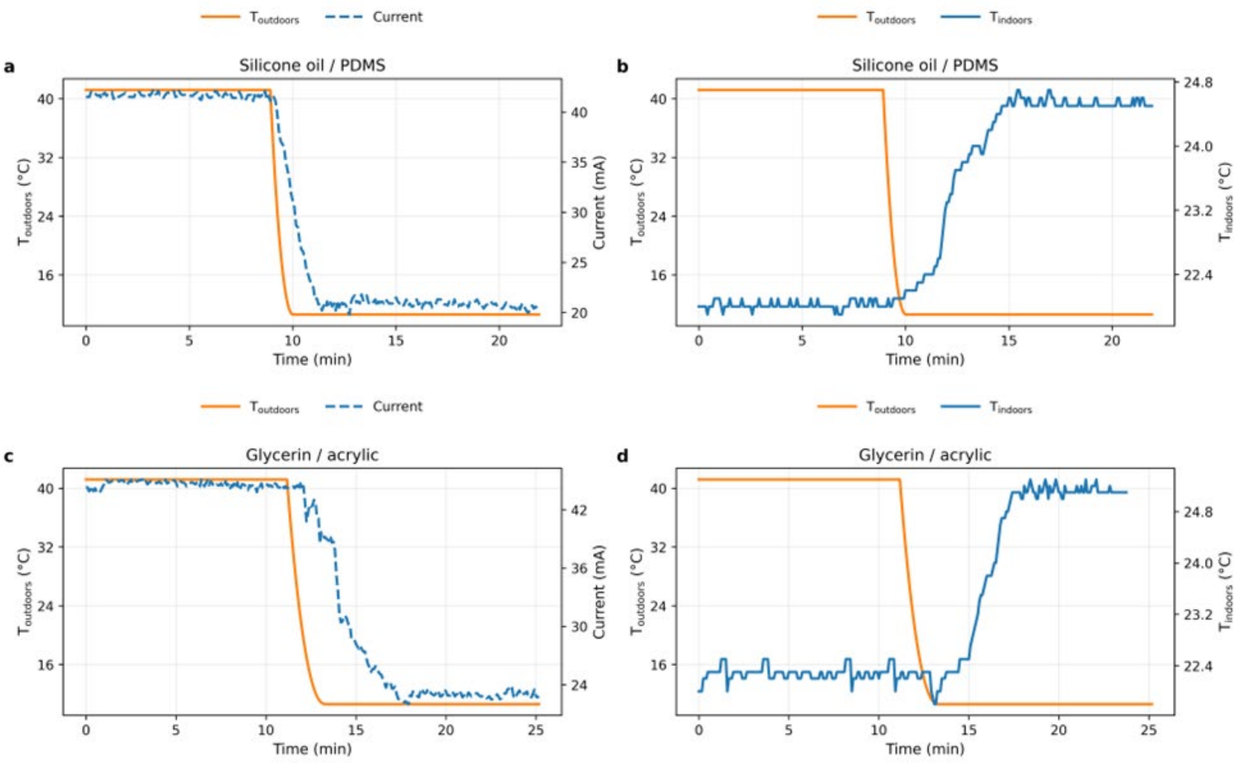


**Figure S15. Generality of the Fresnel waveguiding principle across different refractive-index-matched fluid-solid pairs beyond the water/resin example.** (**a–b**) As a first example, showing consistent dependence between PV signal (current) on outdoor temperature, and inverted dependence between indoor temperature on outdoor temperature, for a device consisting of a Polydimethylsiloxane (PDMS) Fresnel lens (RI ~1.41; Sylgard 184, DOW Silicones Corporation) with an actively flowed silicone oil layer above it (RI ~1.41; Sigma Aldrich, 378364l). (**c–d**) As a second example, showing consistent dependence between PV signal (current) on outdoor temperature, and inverted dependence between indoor temperature on outdoor temperature, for a device consisting of a acrylic/Polymethylmethacrylate (PMMA) Fresnel lens (RI ~1.49) with an actively flowed glycerol layer above it (RI ~1.47). Across both example systems, the PV current and indoor temperature change with the imposed outdoor temperature cycle, consistent with the same refractive-index switching mechanism observed for water/resin-based devices. The experimental set up used to collect this data can be found in Figure 2a and is discussed further in the Materials and Methods section. An Arduino Uno R3 was connected to the PV cell to collect current data.

**Supplementary Note 8: Light intensity distribution.**

**Figures S16 and S17** show the distribution of light intensity measured across the Fresnel lens surface (5 cm diameter; further details in the Materials and Methods section).

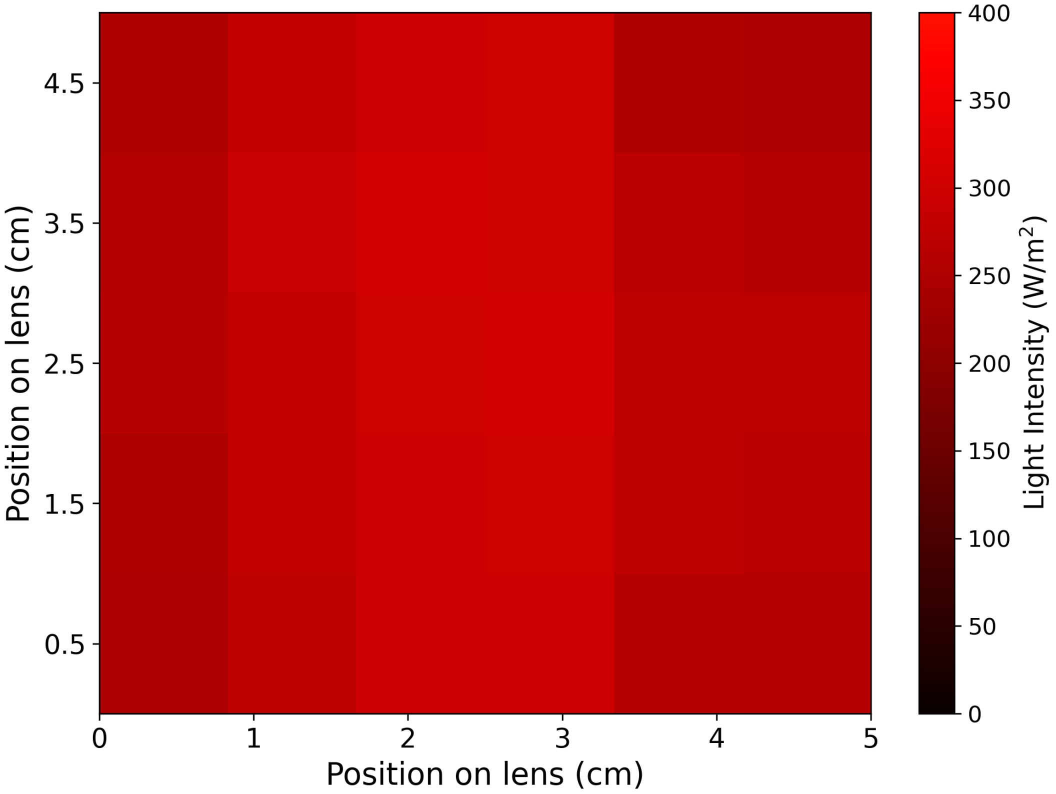


**Figure S16. Measured light intensity distribution across the Fresnel lens surface at 300 W/m$^2$.** Light intensity (W/m$^2$) recorded across the 5-cm-diameter lens surface, for light incident on the lens at 0°, demonstrating uniform, collimated illumination. The light intensity data corresponds to average measurements over three trials.

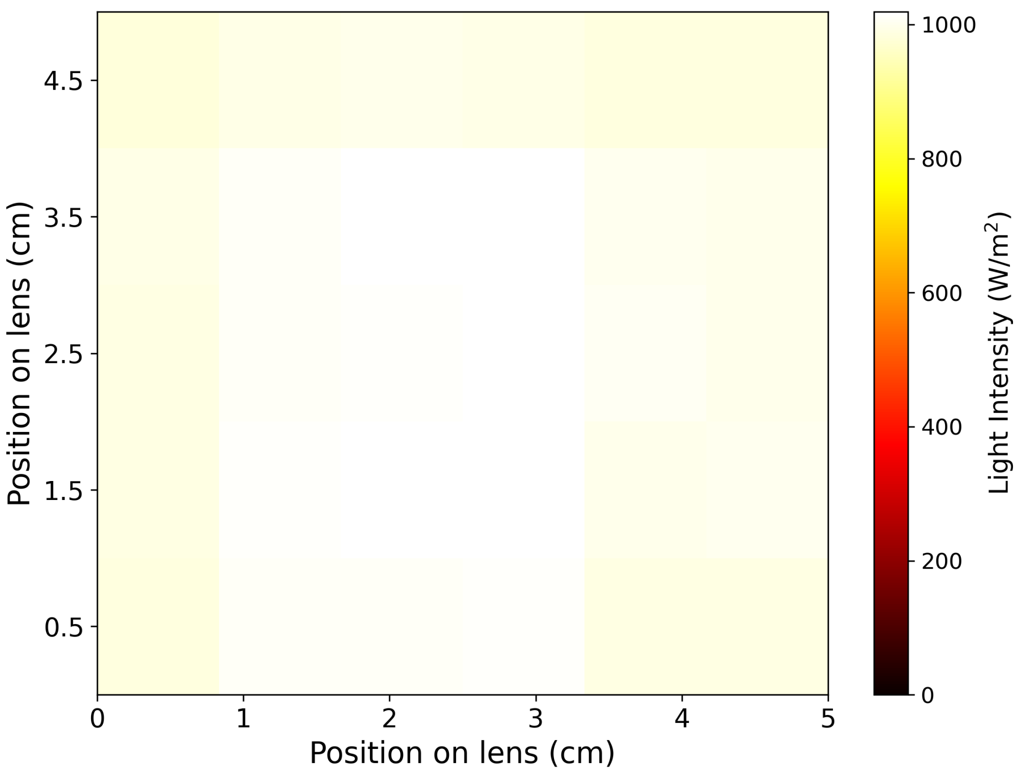


**Figure S17. Measured light intensity distribution across the Fresnel lens surface at 1000 W/m².** Light intensity (W/m²) recorded across the 5-cm-diameter lens surface, for light incident on the lens at 0°, demonstrating uniform, collimated illumination. The light intensity data corresponds to average measurements over three trials.
